# Exertional heat stroke on fertility, erectile function, and testicular morphology in male rats

**DOI:** 10.1038/s41598-021-83121-3

**Published:** 2021-02-11

**Authors:** Pei-Hsuan Lin, Kuan-Hua Huang, Yu-Feng Tian, Cheng-Hsien Lin, Chien-Ming Chao, Ling-Yu Tang, Kun-Lin Hsieh, Ching-Ping Chang

**Affiliations:** 1 Department of Obstetrics and Gynecology, Da-An Women and Children Hospital, Tainan, Taiwan; 2 Division of Urology, Department of Surgery, Chi-Mei Medical Center, Address: No. 901, Zhonghua Rd, Yongkang District, Tainan, 710 Taiwan; 3 Division of Colorectal Surgery, Department of Surgery, Chi Mei Medical Center, Tainan, 710 Taiwan; 4 Department of Medicine, Mackay Medical College, New Taipei, Taiwan; 5 Department of Medical Research, Chi Mei Medical Center, Address: No. 901, Zhonghua Rd, Yongkang District, Tainan, 710 Taiwan; 6 Department of Intensive Care Medicine, Chi Mei Medical Center, Liouying, Tainan, Taiwan; 7 Department of Nursing, Min-Hwei College of Health Care Management, Tainan, Taiwan; 8 Department of Environmental and Occupational Health, College of Medicine, National Cheng Kung University, Tainan, Taiwan

**Keywords:** Reproductive biology, Environmental sciences, Urology

## Abstract

The association of exertional heat stroke (EHS) and testicular morphological changes affecting sperm quality, as well as the association of EHS and hypothalamic changes affecting sexual behavior, has yet to be elucidated. This study aimed to elucidate the effects of EHS on fertility, erectile function, and testicular morphology in male rats. Animals were exercised at higher room temperature (36 ℃ relative humidity 50%) to induce EHS, characterized by excessive hyperthermia, neurobehavioral deficits, hypothalamic cell damage, systemic inflammation, coagulopathy, and multiple organ injury. In particular, EHS animals had erectile dysfunction (as determined by measuring the changes of intracavernosal pressure and mean arterial pressure in response to electrical stimulation of cavernous nerves). Rats also displayed testicular temperature disruption, poorly differentiated seminiferous tubules, impaired sperm quality, and atrophy of interstitial Leydig cells, Sertoli cells, and peri-tubular cells in the testicular tissues accompanied by no spermatozoa and broken cells with pyknosis in their seminal vesicle and prostatitis. These EHS effects were still observed after 3 days following EHS onset, at least. Our findings provide a greater understanding of the effect of experimentally induced EHS on masculine sexual behavior, fertility, stress hormones, and morphology of both testis and prostate.

## Introduction

One of the direct public health risks posed by climate change is increased heat-related mortality and morbidity. The most common heat-related illness is heat exhaustion and may progress to heat stroke, which is a severe illness. Heat stroke is clinically diagnosed as excessive hyperthermia, a central nervous system (CNS) dysfunction, and a history of environmental heat exposure (classic) or vigorous physical activity (exertional)^1,2^. Classic heat stroke is observed primarily in very young and elderly individuals with exposure to hot environments in the absence of strenuous physical activity. Exertional heat stroke (EHS) is observed primarily in healthy young and physically fit individuals (e.g., athletes, firefighters, agricultural workers, soldiers, and football players) that collapse during strenuous physical activity prolonged period in a hot environment^2,3^. EHS can also occur in many kinds of industrial jobs, which are carried out in hot work environments (e.g., steel, glass, ceramics factory, construction, kitchens, laundries, etc.).

The mammalian scrotal temperature is 2–8 °C lower than the core body temperature^4^. Mild scrotal heat stress eliminates the spermatogonial germ cells in the seminiferous tubules and results in decreased sperm density, testicular tissue morphological changes, and infertility^5,6^. Increased scrotal temperatures from occupational exposure, or lifestyle (e.g., prolonged sitting or driving wearing, sauna or steam room user tight-fitting underwear), may lead to male infertility^7,8^. However, the precise role of scrotal hyperthermia associated with infertility remains to be studied. Although numerous studies have investigated the effect of a classic heat stroke on testicular tissue morphological changes affecting the sperm production process as well as infertility^9–11^, the association of EHS and testicular morphological changes affecting sperm maturation as well as the association of EHS and hypothalamic functional changes affecting sexual behavior^12^ has yet to be elucidated in male rats.

A more recent systemic review describes that the incidence of EHS ranged from 0.2 to 10.5 person-years, while the prevalence rates from 0.3 to 9.3% among military personnel^13^. EHS was featured with loss of consciousness, elevated mean core temperature (40–41.6 °C), and elevated levels of creatine phosphokinase, liver enzymes, and creatinine^13^. In this study, we first established a new model in untrained adult laboratory male rats exercising near room temperature that could be used to induce survivable EHS. In EHS, rats exercised in heat until they reached limiting neurological symptoms (loss of consciousness). The symptom-limited maximum core temperatures achieved were 42.9 ± 0.1 °C at 50% relative humidity. All rats that were followed for 3 days survived. Plasma levels of proinflammatory cytokines, disseminated intravascular coagulation (DIC) indicators, and multiple organ damage markers were significantly elevated compared with matched normal controls. Indeed, herein we first reported a new model of survivable EHS in the rat that fully mimicked the structure, functional and biochemical characteristics seen in humans. Second, we assessed the effects of EHS on scrotal temperature, plasma levels of testosterone, sperm viability, and histology of testes, epididymis, seminal vesicle, and prostate. In addition, immediately right after the onset of EHS or 3 days after the onset of EHS, sexual behavioral ability (or penis erectile function) was evaluated in rats as detailed previously^14^. Upon understanding the relationship between EHS and reproduction capacity, it would be to use it as a biomarker for problems with recovery from heat stroke in males.

## Results

### Body core temperature (Tco) and scrotal temperature elevation after EHS onset

**Figure 1:**
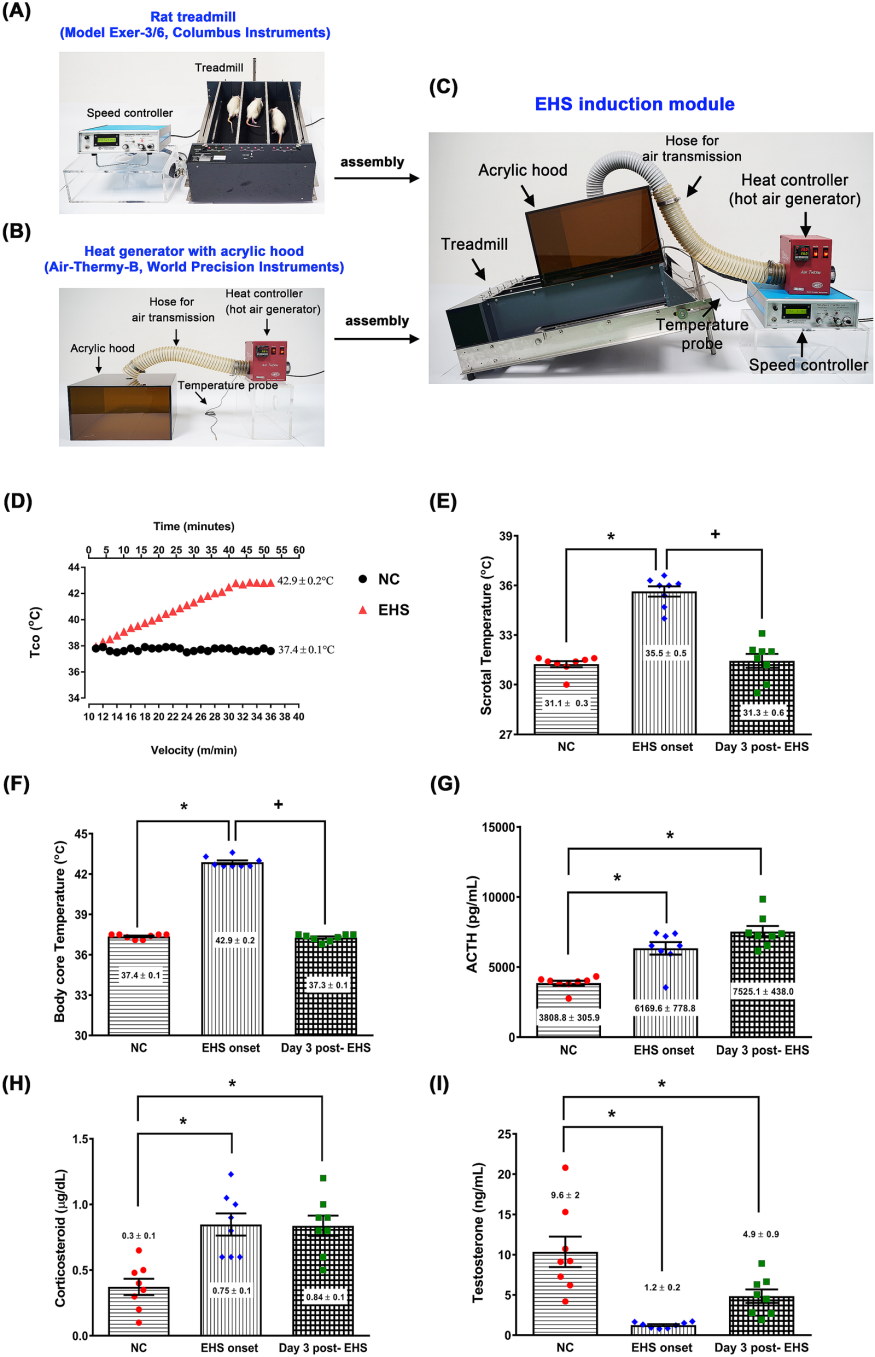
The experimental design. The customized EHS module comprises (**A**) an animal treadmill and is covered with (**B**) an acrylic hood (climatic chamber) that connects the air transmission tube with a hot air generator and temperature monitor. (**C**) The climatic chamber was kept the environmental temperature at 36 °C and 50% relative humidity. The treadmill velocity was increased by 1 m/min every 2 min (constant slope of 15° and an initial velocity of 10 m/min) until each rat has run to exhaustion under the customized EHS module. (**D**) Time to exhaust and the time-dependent and velocity-dependent colonic (core) temperature changes were recorded. The values of body core temperature (**E**), scrotal temperature (**F**), and the values of plasma ACTH (**G**), corticosterone (**H**), and testosterone concentrations (**I**) of rats from the three different groups. Data are presented as the mean ± standard deviation (n = 8 for each group). *P < 0.05, EHS onset vs. NC or Day 3 post-EHS vs. NC. ^+^P < 0.05, Day 3 post-EHS vs. EHS onset.

Figure 1E,F show the values of both body core temperature and rats' scrotal temperature in the different experimental groups. Average values of both temperatures were observed in the non-exercised and non-heated NC group. Compared to the NC group, the EHS onset group, but not the Day 3 post-EHS group, had significantly higher values of both body core temperature (42.9 °C vs. 37.1 °C) and scrotal temperature (35.3 °C vs. 31.2 °C).

### Increased plasma levels of stress hormones, multiple organ damage indicators, DIC indicators and proinflammatory cytokines after EHS onset

The NC group rats show normal plasma levels of stress hormones (ACTH and corticosterone) (Fig. 1G,H), multiple organ damage indicators (BUN, uric acid, creatinine, ALT, AST, and alkaline phosphatase), DIC indicators (APTT, protein C, platelet, and d-dimer), and proinflammatory cytokines (IL-1β, IL-6, and TNF-α) in the NC group (Table 1). However, compared to the NC group, the EHS onset group or Day 3 post-EHS group had significantly higher plasma levels of all these parameters (Fig. 1G,H and Table 1). Figure 1I shows plasma levels of testosterone of rats in different experimental groups. Compared to the NC group, both the EHS onset group and Day 3 post-EHS group had significantly lower testosterone values in their plasma (P < 0.05 and P < 0.01, respectively).

**Table 1:** The mean ± S.D. values (n = 8 for each group) of cardiac injury markers, kidney injury markers, liver injury markers, and disseminated intravascular coagulation (DIC) markers for NC, EHS onset, and Day 3 post-EHS.

Parameters	NC	EHS onset	Day 3 post-EHS
**Cardiac injury markers**			
Creatine kinase-MB (IU/L)	1054 ± 155	3587 ± 363*	3469 ± 387*
Lactate dehydrogenase (IU/L)	918 ± 152	1546 ± 110*	1439 ± 120*
Cardiac troponin I (ng/mL)	0.6 ± 0.1	1.8 ± 0.4*	2.9 ± 0.3*
Myoglobin (ng/mL)	80 ± 18	1492 ± 370*	1377 ± 459*
**Kidney injury markers**			
BUN (mg/dL)	9.8 ± 1.1	38.4 ± 1.9*	37.2 ± 1.6*
Uric acid (mg/dL)	2.2 ± 0.2	4.9 ± 0.2*	4.6 ± 0.2*
Creatinine (mg/dL)	0.6 ± 0.1	1.1 ± 0.1*	1.0 ± 0.1*
**Liver injury markers**			
AST/SGOT (U/L)	105 ± 1	325 ± 8*	307 ± 6*
ALT/SGPT (U/L)	44 ± 3	105 ± 9*	101 ± 8*
Alkaline phosphatase (U/L)	68.9 ± 11	135.7 ± 16*	114.9 ± 15*
**Disseminated intravascular coagulation (DIC) indicators**			
APTT (s)	28 ± 3	92 ± 10*	85 ± 9*
Protein C (μg/L)	3.0 ± 0.4	0.5 ± 0.1*	0.9 ± 0.2*
Platelet count (10^3^/mL)	136 ± 11	52 ± 5*	87 ± 7*
d-dimer (pg/L)	30 ± 2	91 ± 8*	72 ± 6*
**Proinflammatory cytokines**			
Interleukin-1β (pg/mL)	112 ± 14	846 ± 102*	658 ± 87*
Interleukin-6 (pg/mL)	144 ± 22	863 ± 115*	664 ± 74*
Tumor necrosis factor (pg/mL)	27 ± 6	901 ± 123*	725 ± 83*

*P < 0.05, EHS onset vs. NC or Day 3 post-EHS vs. NC.

### Neurological injury after EHS onset

**Figure 2:**
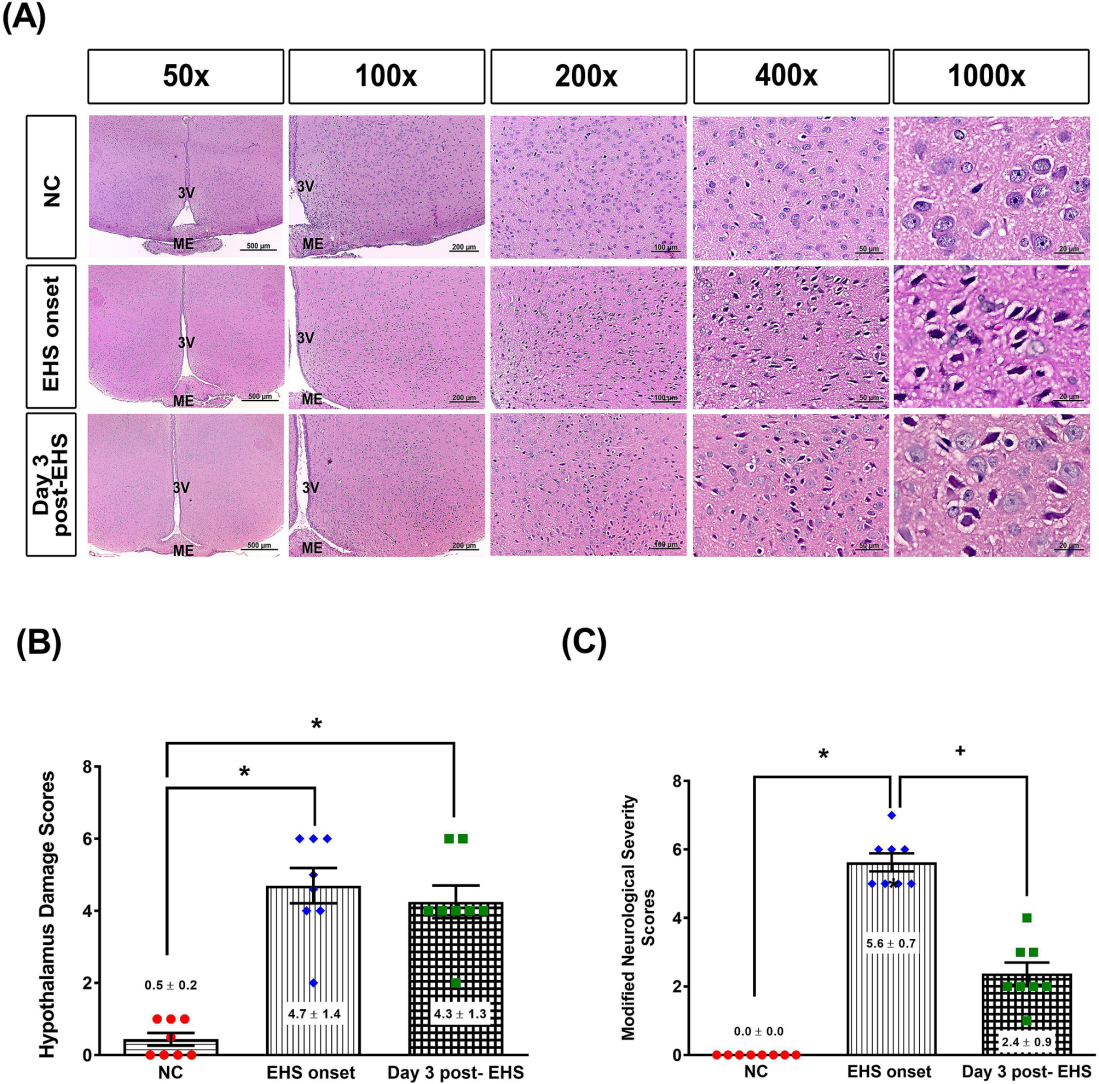
Histological alterations and quantitative analysis of rat hypothalamic tissues and neurological function deficits from the three groups. (**A**) Representative histological images are presented, following hematoxylin and eosin staining of the brain of rats from the NC, EHS onset, and Day 3 post-EHS. Images of brain morphology are shown at ×50, ×100, ×200, ×400 and ×1000 magnification. Scale bars = 500 μm, 200 μm, 100 μm, 50 μm, and 20 μm. (**B**) The quantitative analysis of the histological alterations in the different groups. (**C**) The extents of neurological injury were evaluated by modified neurological severity scores from the three groups. Data are presented as the mean ± standard deviation (n = 8 for each group). The EHS rats displayed many pyknotic cells and vacuolization in their hypothalamic brain tissues. *P < 0.05, EHS onset vs. NC or Day 3 post-EHS vs. NC. ^+^P < 0.05, Day 3 post-EHS vs. EHS onset. 3 V = third ventricle; ME = median eminence.

H&E staining revealed that both the EHS onset group and Day 3 post-EHS group exhibited significantly higher hypothalamic cell damage scores than did the NC group (Fig. 2A). The NC group exhibited almost normal morphology, whereas in the EHS onset and Day 3 post-EHS groups, ~ 50% of the field showed moderate damage (e.g., structural disorganization, edema, many pyknotic cells, and vacuolization). Structural disorganization included numerous degenerating neurons (characterized by brightly stained eosinophilic cytoplasm and dark condensed nuclei). The extent of the neurological injury was evaluated by both hypothalamic damage scores (Fig. 2B) and modified neurological severity scores (mNSS) (Fig. 2C). Both normal morphology and “0” mNSS were observed in the NC group. Compared to NC group, the EHS onset group or Day 3 post-EHS group had significantly higher hypothalamic damage scores (2 vs. 0) and mNSS (5.0 vs. 0.0). The EHS rats displayed moderate damage, including structure disorganization, edema, pyknotic cells, vacuolization, and inflammatory cell infiltration in their brain tissues. They also displayed mild motor, sensory, balance, and reflexes movement deficits based on their performance in the mNSS test.

### Histology of testes after EHS onset

**Figure 3:**
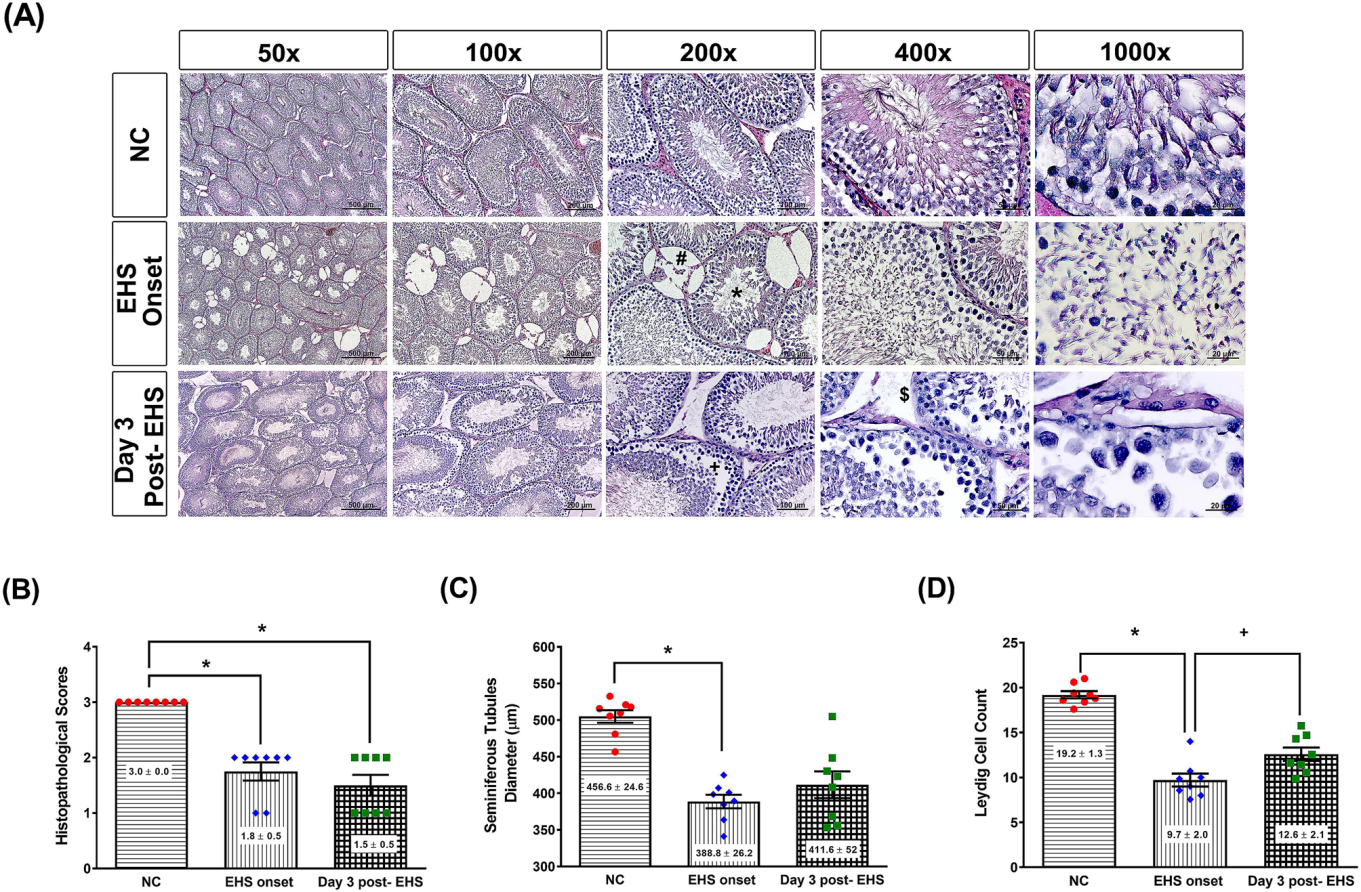
Histological alterations and quantitative analysis of rat testicular tissues from the three groups. (**A**) Representative histological images are presented, following hematoxylin and eosin staining of the testes of rats from the NC, EHS onset, and Day 3 post-EHS. Images of testes morphology are shown at ×50, ×100, ×200, ×400 and ×1000 magnification. Scale bars = 500 μm, 200 μm, 100 μm, 50 μm, and 20 μm.The lower panel denotes a quantitative analysis of the histological alterations of the testes (**B**), the seminiferous tubules diameters (**C**), and the Leydig cell count (**D**) in the different groups. The EHS rats had a reduction of spermatogenic cells (block star), Leydig cells, Sertoli cells, and peritubular myoid cells in their testicular tissues. Data are presented as the mean ± standard deviation (n = 8 for each group). *P < 0.05, EHS vs. NC or Day 3 post-EHS vs. NC. ^+^P < 0.05, Day 3 post-EHS vs. EHS onset.

Figure 3A shows the photomicrographs of the testes of rats in different experimental groups. Seminiferous tubules and spermatozoa populations were normal in the non-exercised and non-heated (NC) group. The EHS onset group or the Day 3 post-EHS group showed poorly differentiated seminiferous tubules, fewer spermatozoa population, and atrophy of adjacent interstitial Leydig cells, Sertoli cells, and peritubular myoid cells. Compare to the NC group, both the EHS onset group or Day 3 post-EHS group had significantly lower values of histopathological scores, seminiferous tubules diameters, and Leydig cell counts (Fig. 3B–D).

### Histology of the epididymis after EHS onset

**Figure 4:**
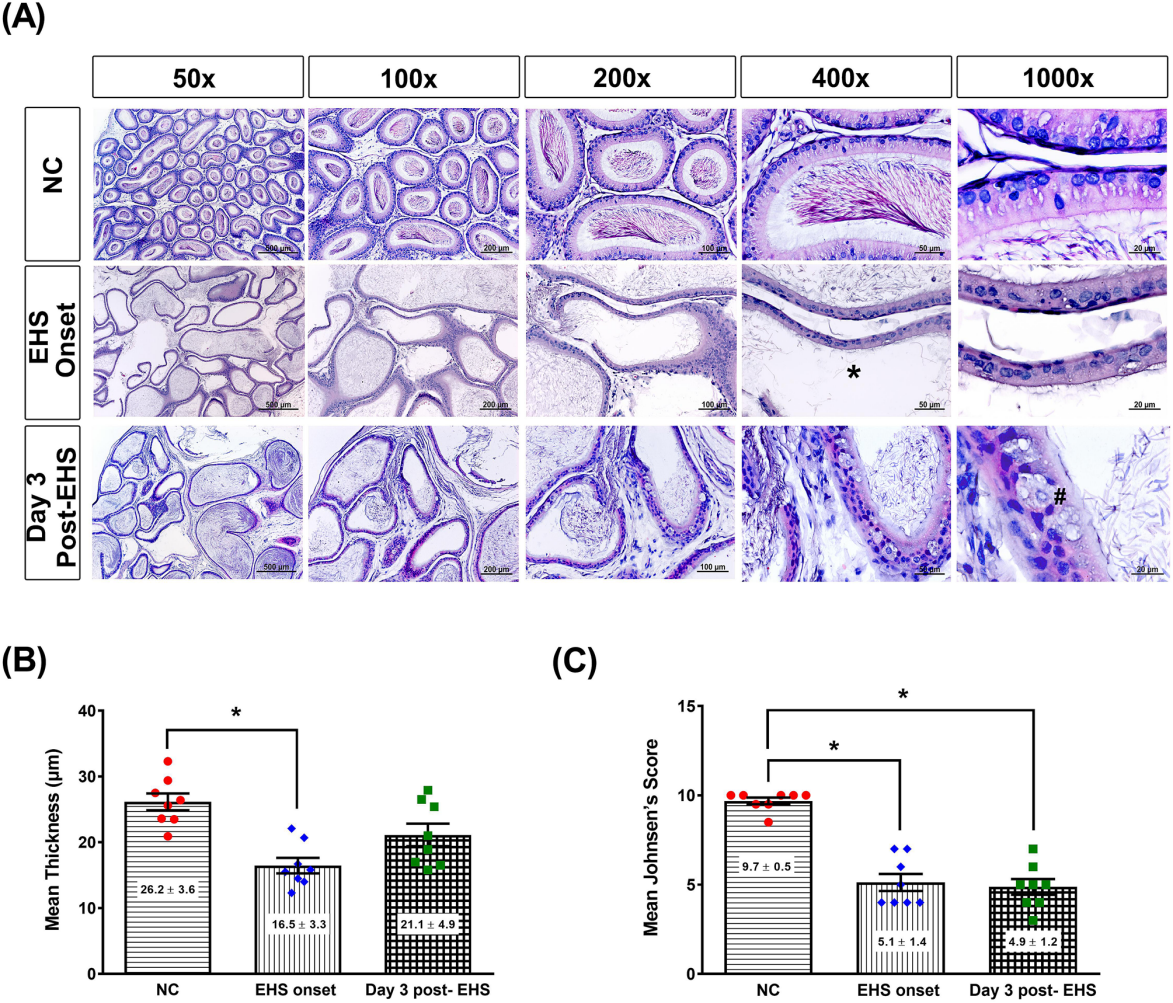
Histological alterations and quantitative analysis of rat epididymis tissues from the three groups. (**A**) Representative histological images are resented, following hematoxylin and eosin staining of the epididymis of rats from the NC, EHS onset, and Day 3 post-EHS. Images of epididymis morphology are shown at ×50, ×100, ×200, ×400 and ×1000 magnification. Scale bars = 500 μm, 200 μm, 100 μm, 50 μm, and 20 μm. (**B**) The mean thickness of the epididymis basement membrane and (**C**) the mean Johnsen’s score were presented as mean ± standard deviation (n = 8 per group). The EHS rats had a reduction of both mean thickness and mean Johnsen’s score of the cytoplasmic membrane, spermatids (#), and cytoplasmic degradation (*). *P < 0.05, EHS onset vs. NC or Day 3 post-EHS vs. NC.

Figure 4A shows a photomicrograph of the epididymis of rats in the different experimental groups. Normal spermatozoa density and intact basement membrane were observed in the non-exercised and non-heated group or NC controls. The EHS onset group or Day 3 post-EHS group shows no spermatozoa and broken basement membranes. Compared to the NC group, both the EHS onset group and Day 3 post-EHS group had significantly higher values of both the mean thickness of the basement membrane (Fig. 4B) and mean Johnsen’s score (Fig. 4C).

### Histology of seminal vesicle after EHS onset

**Figure 5:**
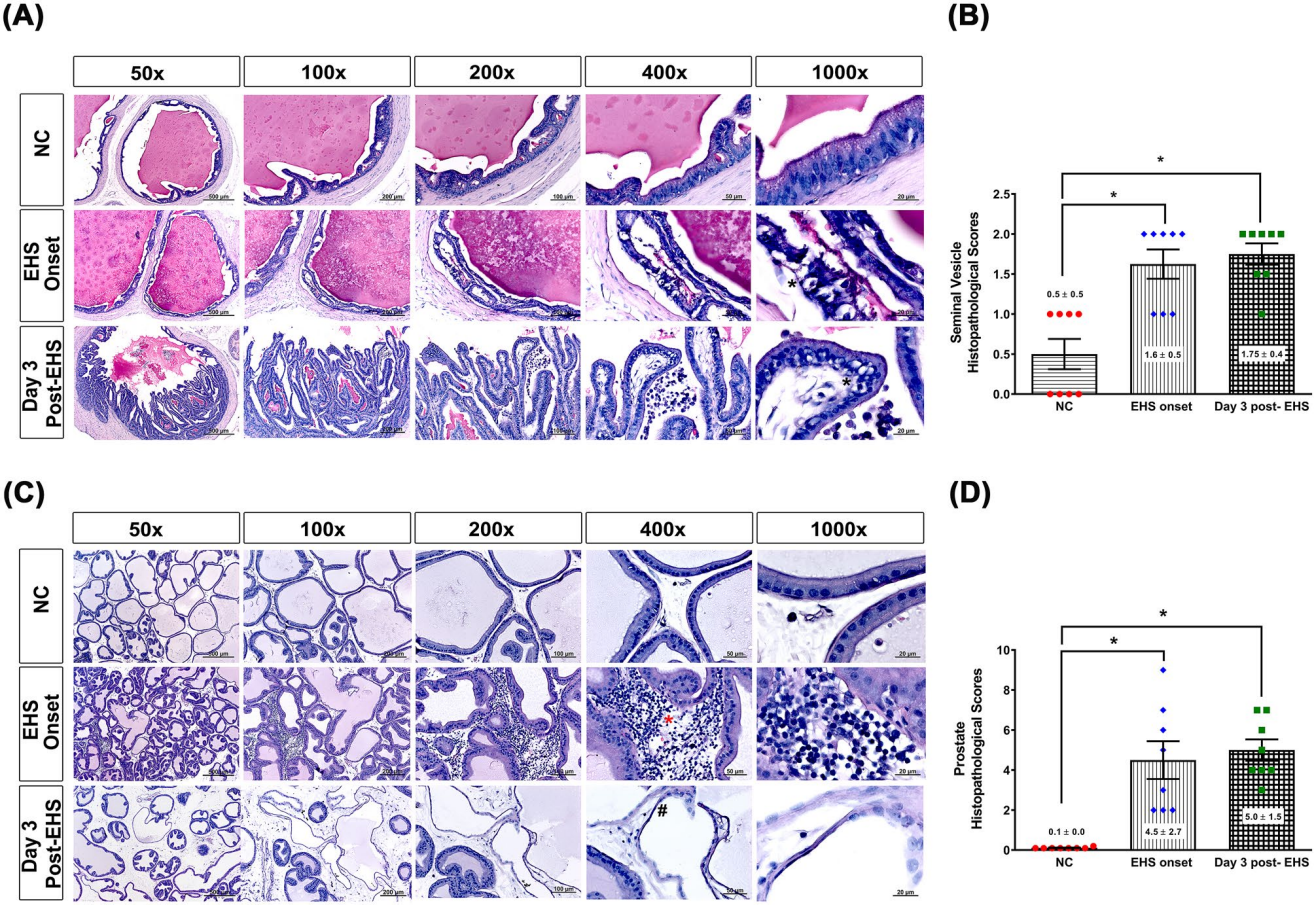
Histological alterations and quantitative analysis of rat seminal vehicle and prostate tissues from the three groups. (**A**) Representative histological images are presented, following hematoxylin and eosin staining of the seminal vesicle of rats from the NC, EHS onset, and Day 3 post-EHS. The EHS rats had many pyknosis cells (black stars) in their seminal vesicle tissues. (**B**) The right panel denotes a quantitative analysis of the histological alterations in the different groups. (**C**) Representative histological images of the prostates of rats from the NC, EHS onset, and Day 3 post-EHS. The red star indicates inflammatory cells in the standard deviation. The red star indicates inflammatory cells in the rats' prostate tissues, and the black # indicates the interstitial edema in the rats' prostate tissues. (**D**) The right panel denotes a quantitative analysis of the histological alterations in the different groups. Images of the seminal vehicle and prostate morphology are shown at ×50, ×100, ×200, ×400 and ×1000 magnification. Scale bars = 500 μm, 200 μm, 100 μm, 50 μm, and 20 μm. Data are presented as the mean ± standard deviation (n = 8 per group). *P < 0.05, EHS onset vs. NC or Day 3 post-EHS vs. NC.

Figure 5A shows photomicrographs of the seminal vesicles of rats in different experimental groups. The cellular structures of seminal vesicles were normal in the NC group. However, the EHS onset group or Day 3 post-EHS group showed the accumulation of degenerative cells with pyknosis in their seminal vesicle. Compared to NC group, both EHS onset group or Day 3 post-EHS group had significantly higher values of histopathological scores (Fig. 5B).

### Histology of prostates after EHS onset

Figure 5C shows the photograph of the prostates of rats in different experimental groups. The cellular structures of prostates were normal in the NC group. However, the EHS onset group or Day 3 post-EHS group had more inflammatory cells and more interstitial edema in the prostate tissues. Compared to NC group, both EHS onset group or Day 3 post-EHS group had significantly higher values of histopathological scores (Fig. 5D).

### Intracavernosal pressure (ICP) recording to evaluate erectile function after EHS onset

Erectile function in rats can be evaluated by measuring the ICP. In practice, ICP can be monitored following electrical stimulation of the cavernous nerves. The mean arterial pressure (MAP) is used as a reference for ICP. Using ICP recording protocols, many key parameters of erectile function can be measured from the ICP response curve^15^.

**Figure 6:**
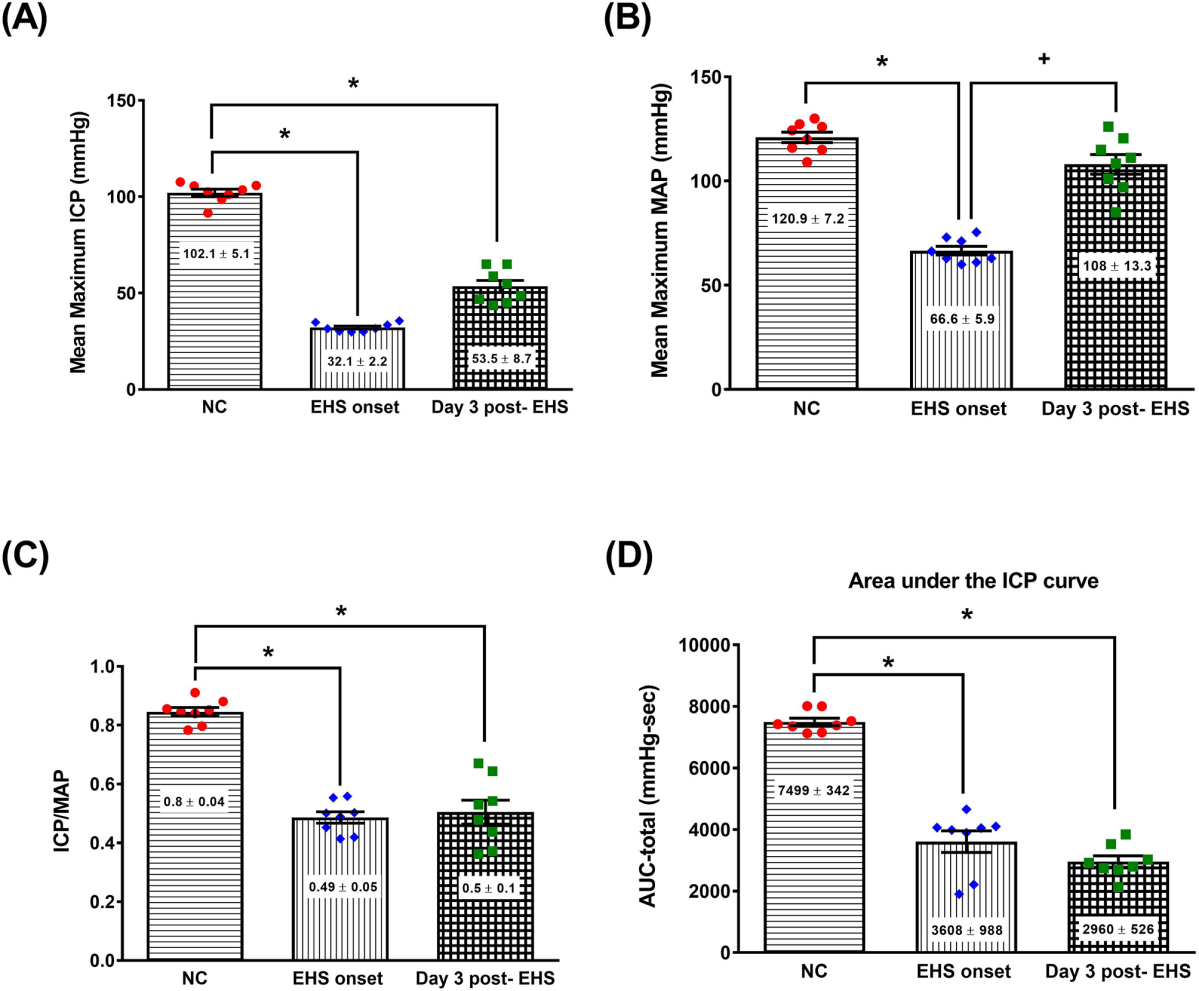
The values of (**A**) mean maximum intracavernosal pressure (ICP), (**B**) mean maximum arterial pressure (MAP), (**C**) ICP/MAP, and (**D**) area under the ICP curve (Auc-total) of rats in different groups. Data are presented as the mean ± standard deviation (n = 8 per group). *P < 0.05, EHS onset vs. NC, or Day 3 post-EHS vs. NC. ^+^P < 0.05, Day 3 post-EHS vs. EHS onset.

Figure 6 shows the mean maximum ICP (Fig. 6A), mean maximum MAP (Fig. 6B), ICP/MAP (Fig. 6C), and area under the ICP curve (AUC-total, Fig. 6D) of rats in different experimental groups. Compared to the NC group, both the EHS onset group or the Day 3 post-EHS group had significantly lower values of ICP, MAP, ICP/MAP, and AUC-total.

### Impairments of sperm quantity and quality after EHS onset

**Figure 7:**
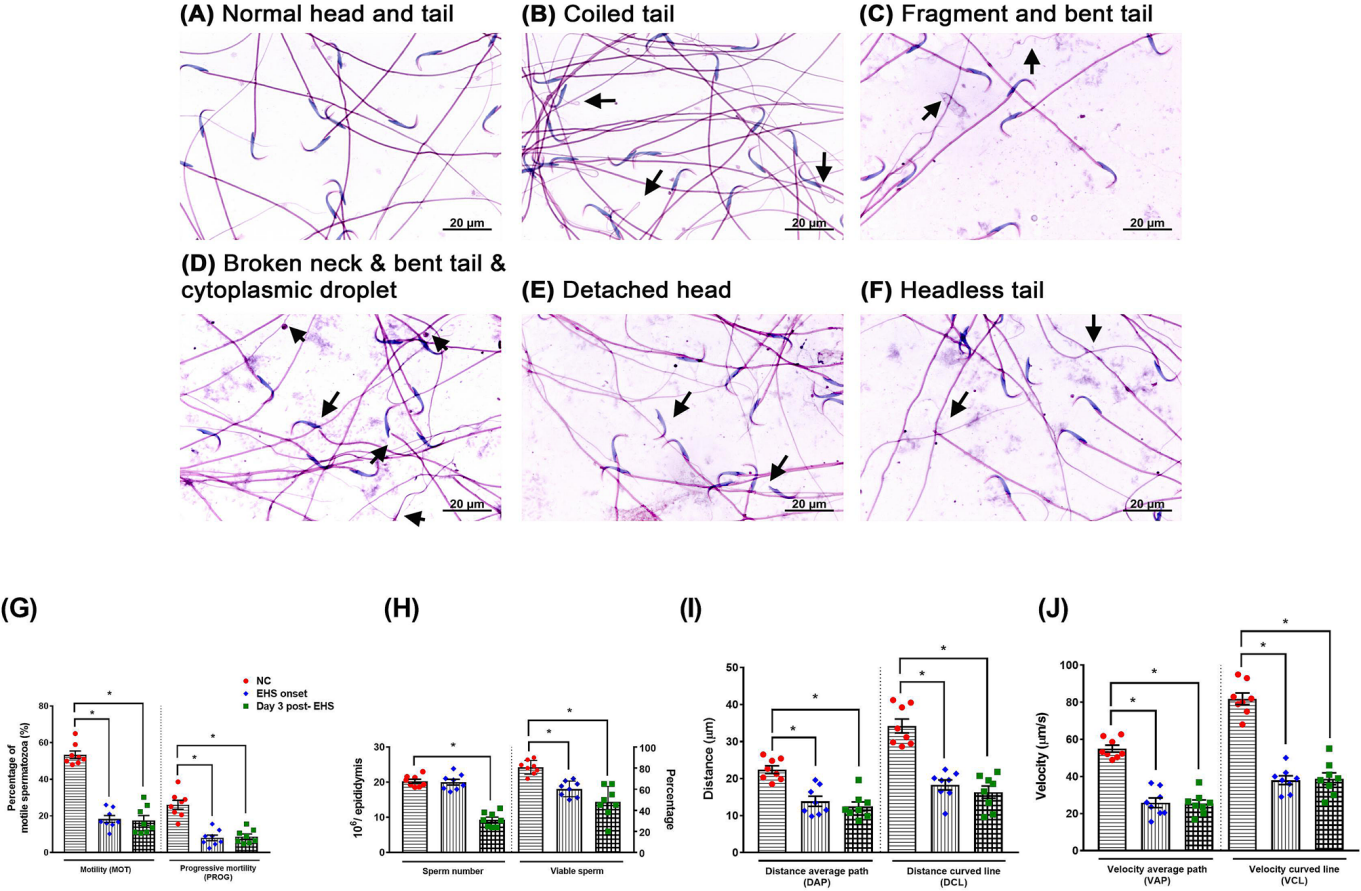
Microphotographs were illustrating morphologically normal sperm and various sperm defects. (**A**) Normal head and tail from a NC rat. (**B**) Coiled and (**C**) fragment and bent tail from an EHS onset rat. (**D**) Broken neck, bent tail, cytoplasmic droplet, (**E**) detached head, and (**F**) headless tail from a Day 3 post-EHS rat. (**G**) The percentage of sperm motility (MOT) and the percentage of sperm with progressive motility (PROG), (**H**) sperm numbers and viable sperm percentages, (**I**) the distance average path (DAP) and distance curved line (DCL), and (**J**) the velocity average path (VAP) and velocity curved line (VCL) were elevated (p < 0.05) in the EHS onset and Day 3 post-EHS group when compared to NC animals. Data were the mean ± standard deviation (n = 8 per group). *P < 0.05, EHS onset vs. NC or Day 3 post-EHS vs. NC.

Compared to the NC group (Fig. 7A), the EHS onset group (Fig. 7B,C) or the Day 3 post-EHS group (Fig. 7D–F) had significantly abnormal morphology. The predominant types of abnormalities were sperm with a bent tail (Fig. 7C), broken neck (Fig. 7D), cytoplasmic droplet (Fig. 7D), detached head (Fig. 7E), and headless tail (Fig. 7F). The presentative microphotographs of sperm morphology at 400× magnifications are shown in Fig. 7A–F. Compared to the NC group, the EHS onset group or Day 3 post-EHS group had a significant decrease in MOT (p < 0.01) and PROG (p < 0.01) (Fig. 7G). Compared to the NC group, the EHS onset group had an insignificant change (p > 0.05) in the sperm numbers but had significant decrease in the percentages of viable sperms (Fig. 7H). The Day 3 post-EHS group had a significant decrease in both the sperm numbers and the percentage of viable sperms (Fig. 7H).The results also showed decreased distance parameters (DAP and DCL, Fig. 7I) and velocity (VAP and VCL, Fig. 7J) (p < 0.01).

## Discussion

Our present study provides a new model in untrained adult male laboratory rats exercising at higher room temperature that mimicked survival EHS in humans. Compared to untrained adult laboratory male rats nonexercising near room temperature (26 °C, RH 50%), rats exercising at higher environmental temperature (36 °C, RH 50%) displayed survival EHS significantly. The EHS reactions included immobilization, higher body temperature, higher neurological functional deficits, higher hypothalamic damage scores, and higher plasma levels of stress hormones, multiple vital organs injury indicators, and disseminated intravascular coagulation (DIC). These EHS reactions lasted at least up to three days post-EHS. All EHS animals that were followed over the 14-day recovery period survived the EHS challenges (data not shown). Heat stress and heat illness arise when ambient temperatures are high. They can be triggered by physical exertion even at low-risk temperature^16^. Physical exertion can lead to heat stress and heat illness (due to intense and rapid heat production in the working muscles). Higher environmental temperatures further increase the risk. Thus, our present model fulfills that EHS is associated with excessive hyperthermia (over 40 °C Tco), encephalopathy, and confusion or coma during or just after strenuous physical activity in young, previously healthy subjects^17^. Indeed, in our rats, EHS occurs within the first 2 h of exertion and not necessarily at high ambient temperatures^18^. Compared to classic heat stroke, the death rate from EHS is relatively low or zero.

Acute exposure (from seconds to a few hours) to stressors such as immobilization^19^, electric foot shocks^20^, cold, ether^21^, exercise^22^, food restriction^23^ or anxiety situations^24^ in males causes an increase in corticotropin-releasing hormone, adrenocorticotropin hormone (ACTH), β-endorphins and corticosterone. In rats, acute exposure to noise or water immersion^25^, immobilization^26^, cold^27^, hot^28^, light^25^ or surgery^29^ stimulates hypothalamus-pituitary–gonadal (HPG) axis and causes an increase of follicle-stimulating hormone (FSH), luteinizing hormone (LH), and testosterone in the plasma of stressed males. Our present results showed that EHS caused by acute heat exposure and exercise (52 min) was accompanied by increased ACTH and increased corticosterone in the plasma and an increase in hypothalamic damage score in stressed male rats. Additionally, erectile function in rats at EHS onset or Day 3 post-EHS can be evaluated by measuring the ICP. It was found that following the onset of EHS, male rats displayed suppression of erectile function, which can be associated with impairments in both hypothalamic–pituitary–adrenal (HPA) axis activity and HPG activity. In the present study, combined heat stress and exercise might suppress masculine sexual behavior via increasing both HPA and HPG activity^30^.

One of the most critical factors predisposing bulls to subfertility in tropical or subtropical countries is the high environmental temperature^31^. Spermatogenesis depends on the maintenance of testicular temperature from 2 to 6 °C below corporal temperature^32^. Indeed, as shown in the present study, EHS can disrupt testicular thermoregulation (increasing the scrotal temperature from the resting level of 31.2 °C to a new level of 35.3 °C), leading to poorly differentiated seminiferous tubules and seminal vesicle, impairments of sperm quality, and atrophy of adjacent interstitial Leydig cells, Sertoli cell, and peritubular cells. Rats with EHS had no spermatozoa and broken basement in their epididymis, and accumulated degenerative cells with pyknosis in their seminal vesicle. EHS effects were stilled observed after 3 days following EHS onset at least. Based on a recent report of Garcia-Oliveros et al.^33^, EHS might first cause an increase in testes morphological defects, followed by increased sperm lipid peroxidation, thereby inducing mitochondrial distress, reduced sperm motility, and sperm DNA fragmentation.

Spermatozoa are produced in the testes and fully matured in the caput epididymis. Several notable factors, such as increased scrotal temperature resulting from occupational exposure lifestyle or cryptorchidism, contribute to male infertility^7^. The present study demonstrates that EHS impairs sperm quality and can reduce male fertility in rats. Limited clinical studies have promoted that transient scrotal temperature disruption of adult human males may result in reversible spermatogenic arrest and could be used as a contraception method^34^.

Prostatitis is the most common urinary disease type in males < 50 years of age^35^. It can be divided into five categories: acute bacterial prostatitis, chronic bacterial prostatitis, chronic prostatitis, chronic pelvic pain syndrome, and asymptomatic inflammatory prostatitis^36^. Chronic non-bacterial prostatitis may cause male infertility and sexual dysfunction^37^. As shown in the present results, EHS induces subepithelial inflammatory infiltration and interstitial edema in rats' prostate tissues^38^. EHS might likely cause subinfertility and sexual dysfunction via inducing acute non-bacterial prostatitis in mice.

Although it is well known that heat exposure impacts the reproduction capacity of bulls, the rationale of the present study would be to use it as a biomarker for problems with long term recovery from that EHS in males. However, we did not look at long term recovery in our present study. It would be very interesting to know if some form of injury was sustained for prolonged periods as this could lead to an outcome viable in humans that would be clinically valuable. Another limitation of the present study is the heavy instrumentation given to the animals before heat stroke exposure and electric shock stress. The animals had a rectal thermistor during the exercise protocol. The elevated glucocorticoids measured in the control animals is likely a reflection of this.

In conclusion, the present study reported that male rats exercising at higher environmental temperatures (36 °C, RH 50%) displayed survival EHS characterized by the neurobehavioral deficit, hypothalamic damage, and HPA axis or HPG axis impairments, systemic inflammation, DIC, and multiple organs dysfunctions. In addition, rats following EHS onset had sexual dysfunction, testicular temperature disruption, poorly differentiated seminiferous tubules, impairments of sperm quality, and atrophy of interstitial Leydig cells, Sertoli cells, and peri-tubular cells in the testes tissues. Rats with EHS had no spermatozoa and broken cells with pyknosis in their seminal vesicle and prostate. These EHS effects were still observed after 3 days following EHS onset, at least. Collectively, the present results provide a greater understanding of the effects of experimentally induced EHS on masculine sexual behavior, fertility, HPA axis activity, and morphology of both testes and prostates.

## Methods

### Animals

Seventy-two male Sprague–Dawley rats, 7 weeks old weighing between 240 and 255 g, were obtained from the colonies of BioLASCo Taiwan CO., Ltd. (Taipei, Taiwan). The rats were housed 4 per cage in an environmental chamber maintained at 24 °C and 50% relative humidity (RH) in Chi Mei Medical Center and identified by a number printed on the tail base. Lighting was controlled automatically from 08:00 a.m. to 08:00 p.m. Both standard laboratory chow and water were provided each day ad libitum. All of the experiments were conducted in daytime conditions under the light. All the environmental protocols were approved by the Institutional Animal Care and Use Committee at Chi Mei Medical Center (IACUC approval no. 106121110). We used the ARRIVE checklist^39^ when writing our report.

### Familiarization

Before experimentation, rats were familiarized with a load-increasing treadmill (TM) running for 7 days. On Day 1–2 of the program, the rats were placed on the TM, got acclimation to the EHS environment, and then walked on the treadmill of their own free will for 10-min. Rats were first accustomed to a 10-min period of TM running (model: Exer-3/6, Columbus Instruments, Columbus, OH, USA) at a speed of 10 m/min, and a grade of 15° daily and consecutively for 2 days at an environmental status with 26 °C ambient temperature and 50% RH. Then, rats were familiarized with a 10-min period of TM running at a speed of 15 m/min and a grade of 15° daily in the next days 5–7. Seventeen percent of rats cannot complete the acclimation phase were excluded.

### Induction of exertional heat stroke (EHS) and experimental procedures

We randomly (computer-generated randomization) divided 72 rats into EHS group (n = 48) and a normothermia control (NC) group (n = 24). The former was kept at a high room temperature of 36 °C ± 1 °C, and an RH of 50%, whereas the latter was maintained at a room temperature of 26 °C ± 1 °C and an RH of 50%. EHS group was further divided into onset group and day 3 post-EHS, each comprising 24 animals.

Forty-eight rats eligible for exercises after training were selected for the induction of EHS. Between 0900 and 1000, a caged rat was transferred from the vivarium to the climatic chamber on the day of each experiment. To measure body core temperature or rectal temperature (Tco), a protective sleeve and thermistor (Yellow Spring Instrument, Yellow Spring, OH, USA) were inserted 5 cm into the rectum and secured with the surgical tape. A half-hour after insertion of the thermometer, rats were exercised to exhaustion on the treadmill (Model Exer-3/6, Columbus Instruments, Columbus, OH, USA; initial velocity: 10 m/min; 15° slope) (Fig. 1A) in a customized acrylic climatic chamber that connects the air transmission tube with hot air generator (Air Therm model Air-Thermy-B, World Precision Instruments, FL, USA) (Fig. 1B), and keeps at 36 °C and 50% relative humidity. EHS was induced by increasing the initial treadmill velocity 1 m/min every 2 min until the rat appeared to be unable to run. Exhaustion was operationally defined as the third time a rat could no longer keep pace with the speed of the treadmill belt and remained on an electric shock grid for 2 s. Time to exhaust and the time-dependent and velocity-dependent colonic temperature changes were recorded under the customized EHS module (Fig. 1C). The increased treadmill velocity, as well as the increased core temperature during EHS. The average exhaustion time was 52 ± 2.6 min, and the core temperature reached 42.9 ± 0.2 °C (Fig. 1D). Once the exhaustion was confirmed, the rat was removed from the treadmill and subjected to neurological severity tests or biochemical or histological evaluation at room temperature. Another 24 rats were designated as normal controls (NC group). They were subjected to room temperature of 26 ± 1 °C with 50% ± 2% relative humidity with a non-exercised status for exactly 52 min. These non-exercised rats maintained their levels of brightness, alertness, and responsiveness throughout the entire experiment. Rats that survived to day 3 of EHS or control experiments were considered survivors.

In experiment 1: Rats’ body core temperature and scrotal temperature were measured. After mNSS tests, rats were euthanized with an overdose of Zoletil (100 mg/kg body weight), obtained the semen for sperm motility and morphology assay, and collected blood samples from the tail vein for biochemical analysis (n = 8 for each group).

In experiment 2: Rats were euthanized with an overdose of Zoletil (100 mg/kg body weight) and perfused with normal saline followed by 10% neutral-buffered formalin via left heart ventricle puncture. Organs were removed and fixed in 4% paraformaldehyde, processed for paraffin embedding (n = 8 for each group).

In experiment 3: Rats were anesthetized with 2% isoflurane (Sigma-Alrich, MA, USA) in nitrous oxide/oxygen (69%/30%) via face mask, the erectile function was evaluated by intracavernosal pressure (ICP) and mean arterial pressure (MAP) ratio (n = 8 for each group).

### Neurological severity scores

We adopted a modified neurological severity score (mNSS) test detailed previously^40^ to evaluate the extent of the motor, sensory, reflex, and balance deficits. These tests are similar to the contralateral neglect tests in humans. Neurological function was graded on a scale of 0 to 18. One point is awarded for the inability to perform the tasks as for lack of a tested reflex: 13–18 points, severe injury; 7–12 points moderate injury; 1–6 points mild injury. Thereafter a scale of 1–6, 7–12, and 13–18 denote mild injury, moderate injury, and severe injury.

### Erectile function evaluation

Immediately right after the onset of EHS or 3 days after the onset of EHS, erectile function was evaluated in anesthetized rats, and the bilateral major pelvic ganglion (MPG) and cavernous nerve (CN) were exposed as detailed previously^14^. Intracavernosal pressure (ICP) and mean arterial pressure (MAP) were measured. For the electrical stimulation of the CN, the stimulation parameters were 2.5 V at a frequency of 15 Hz with a square wave duration of 1.2 ms for 1 min. The ratio of the maximal ICP to the corresponding MAP (ICP/MAP) was calculated and recorded. In addition, the mean maximum ICP and the total ICP of the tumescence determined by the area under the total ICP curves (AUC) from the beginning to the end of the CN stimulation (60 s) were recorded. The electrical stimulation was always done in triplets with a 5-min interval between the subsequent stimulations to ensure stable activity in every rat.

### Determination of sperm motility and morphology

Sperm samples were taken from the cauda epididymis. Place the cauda in a petri dish containing 10 ml PBS prewarmed to 37 °C, and mince using two no.11 scalpel blades to open the epididymal duct and release its contents. Swirl the petri dish several times to achieve a uniform sperm suspension. For sperm counting, 10 μl of the sperm suspension was placed on the Neubauer hemocytometer, allowed to sediment by standing for 5 min. Place the hemacytometer on the microscope stage and the number of spermatozoa in five squares was counted. The spermatozoa viability was determined using a colorant constituted by nigrosin, eosin, and sodium citrate, all dissolved in distilled water. Ten μl of semen with 10 μl of the colorant were placed on a microscope slide. The stained spermatozoa were considered as dead and those not stained as alive.

The motion parameters included the percentage of motile spermatozoa (MOT), the percentage of progressive motility (PROG), distance average path (DAP, μm), distance curved line (DCL, μm), velocity average path (VAP, μm/s), and velocity curved line (VCL, μm/s) from the epididymis sperm samples were determined within 2–4 min after sacrificed following the method of previous studies^41,42^ and analyzed by IVOS CASA system (Hamilton Thorne, Inc., Beverly, MA, USA) under Zeiss microscope (Carl Zeiss Microscopy GmbH, Jena, Germany). Sperm samples were fixed with Hancock’s solution and stained with Giemsa’s dye for sperm morphology analysis^42^.

### Evaluation of plasma hormones concentration

Plasma levels of testosterone, adrenocorticotropic hormone (ACTH), and cortisol were determined using commercialized immunoassay kits according to the manufacturer’s instructions. The sensitivity of the testosterone assay (ABBOTT ARCHITECT i2000 SR analyzer, Wiesbaden, Germany) was 0.07 ng/ml and with negligible cross-reactivity with other androgen derivatives. The intra-assay coefficients of variation for the testosterone assay were 4.1%. Plasma ACTH and cortisol were assayed using radioimmunoassay kits (CisBio Bioassays, Bagnol sur Cèze, France) with Gammar-counter (PerkinElmer Inc., MA, USA) according to the manufacturer’s protocol.

### Biochemical estimation

At particular time points, rats were anesthetized by giving 80 mg/kg ketamine and 5 mg/kg xylazine intraperitoneally. Whole blood (7 mL) was obtained from the heart puncture and collected into sodium citrate tubes for plasma. The plasma levels of activated partial thromboplastin time (APTT), protein C, platelet count, and d-dimer were measured by automated coagulation instruments (Werfen ACL TOP350, Bedford USA). The platelet counts were measured by automated blood cell counting instruments (Sysmex XN, Wakinohama, Japan). To assess the renal, hepatic, and cardiac functions, we determined the plasma concentration of blood urea nitrogen (BUN), uric acid, creatinine, aspartate aminotransferase (AST), alanine aminotransferase (ALT), alkaline phosphate, creatine kinase-MB (CK-MB), cardiac troponin-I, lactate dehydrogenase, and myoglobin by chemistry analyzer (ABBOTT ARCHITECT c8000/c16000, Illinois, USA). Protein C in the sample was activated by a specific snake venom activator. The resulting protein C activator was assayed in a kinetic test by measuring the increase in absorbance at 405 nm. The reagents for the determination of protein C activity were provided by Berichrom Protein C (Dade Behring Maberg Gm 6H/Marburg, Germany).

For the determination of tumor necrosis factor-alpha (TNF-α; #558535, BD Biosciences, CA, USA), interleukin-1beta (IL-1β; #DY501, R & D system, MN, USA), and interleukin-6 (IL-6; #550319, BD Biosciences, CA, USA), the supernatants were stored at − 70 °C until measurement. They were determined using a double-antibody sandwich enzyme-linked immunosorbent assay.

### Histopathological studies

We got the histological measures and others at the time point of EHS onset in rats and at 3 days after EHS by killing extra groups of rats. Testis tissues fixed in 10% neutral buffered formalin were processed for routine histological preparations. Histopathological assessment of the testes and epididymis was done using the hematoxylin and eosin (H & E) technique. Sections of the right testes and epididymis of each rat were examined for seminiferous tubules diameters and number of Leydig cells in 20 random intertubular regions can area surrounded by three seminiferous tubules using a light microscope at a magnification of 400×. Mean Johnsen’s testicular biopsy score^43^ was assessed in 10 seminiferous tubules. Based on the report of Ebokaiwe et al.^41^, the histopathological scores for testicular damage was measured and recorded.

The brain was removed, fixed in 10% neutral buffered formalin, and embedded in paraffin blocks. Serial (10 μm) sections through the hypothalamus were stained with hematoxylin and eosin for microscopic evaluation. The extent of hypothalamic damage was scored on a scale of 0–3, modified from the previous^44^ grading system.

### Statistical analysis

The person charged with functional outcome measurements was the only one blinded to experiments among those working on animals (single-blind). She used animal codes to recognize individuals and to report repeated measurements on data collection forms. Data are presented as the mean ± S.D. For analysis of physiological parameters (core body temperature and scrotal temperature), behavior parameters (mNSS), hormone data (ACTH, corticosteroid, and testosterone), and biochemical data (BUN, AST, ALT etc.), we performed one-way ANOVA followed by Tukey's post hoc test. Parameters such as histological scores with non-normal distribution were analyzed by the Kruskal–Wallis test with Dunn’s post-hoc test. We used GraphPad Prism (version 7.01 for Windows; GraphPad Software, San Diego, CA, USA) to analyze the data and set the statistically significant level at P < 0.05.

### Ethics approval

All animal experiments were conducted under protocols approved by the Institutional Animal Care and Use Committee of Chi Mei Medical Center, Tainan, Taiwan (approved no.: 108120117) in accordance with the guidelines of Guide for the Care and Use of Laboratory Animals published by the US National Institutes of Health with due consideration to minimize pain and suffering.

## Data Availability

The datasets used and/or analyzed during the current study are available from the corresponding author on reasonable request.

## Abbreviations

EHS: Exertional heat stroke
DIC: Disseminated intravascular coagulation
RH: Relative humidity
NC: Normal control
Tco: Core temperature
TM: Treadmill running
ICP: Intracavernosal pressure
MAP: Mean arterial pressure
CN: Cavernous nerve
AUC: Area under the total ICP curve
mNSS: Modified neurological severity score
MOT: Percentage of motile spermatozoa
PROG: Percentage of progressive motility
DAP: Distance average path
DSL: Distance curved line
VAP: Velocity average path
VCL: Velocity curved line
ACTH: Adrenocorticotropic hormone
FSH: Follicle-stimulating hormone
LH: Luteinizing hormone
APTT: Activated partial thromboplastin time
BUN: Blood urea nitrogen
AST: Aspartate aminotransferase
ALT: Alanine aminotransferase
CK-MB: Creatine kinase-B
TNF-α: Tumor necrosis factor-alpha
IL-1β: Interleukin-1 beta
IL-6: Interleukin-6
H & E: Hematoxylin and eosin
CRH: Corticotropin-releasing hormone
HPA: Hypothalamus–pituitary–adrenal
HPG: Hypothalamus–pituitary–gonadal
